# Differential Responsiveness of Human Skin Mast Cells to SCF and IL-33: Reduced Reactivity to SCF but Not to IL-33 in the Post-Mitotic Phase

**DOI:** 10.3390/cells15050398

**Published:** 2026-02-24

**Authors:** Manqiu Jin, Jean Schneikert, Anja Wegner, Torsten Zuberbier, Magda Babina

**Affiliations:** 1 Institute of Allergology, Charité—Universitätsmedizin Berlin, Corporate Member of Freie Universität Berlin and Humboldt Universität zu Berlin, 12203 Berlin, Germany; manqiu.jin@charite.de (M.J.); jean.schneikert@charite.de (J.S.); anja.wegner@charite.de (A.W.); torsten.zuberbier@charite.de (T.Z.); 2 Fraunhofer Institute for Translational Medicine and Pharmacology ITMP, Immunology and Allergology IA, 12203 Berlin, Germany

**Keywords:** mast cells, skin, SCF, IL-33, signal transduction, immediate-early genes, cytokines, TNF, LIF, IL-13

## Abstract

Skin mast cells (MCs) play a vital role in acute allergic reactions and also contribute to chronic dermatoses partially through cytokine production. Key growth factors (GFs), such as SCF and IL-33, orchestrate MC survival and activity. Whether early responses differ between these factors remains incompletely defined. In the skin, MCs are long-lived and can proliferate outside the body but eventually exit the cell cycle. It remains unclear whether post-mitotic MCs show altered sensitivity to GFs. MCs were isolated from human foreskin and cultured in the presence of SCF + IL-4. GF-deprived cells were stimulated with either SCF or IL-33. Signaling events were determined by immunoblot. Gene expression was studied by RT-qPCR, cytokine release by ELISA, comparing dividing (3–4 weeks) with post-mitotic “aged” MCs (≥6 weeks). SCF strongly induced genes like *FOS*, *EGR1*, and *NR4A2*, while IL-33 was particularly effective at inducing *JUN*. IL-33 also prompted significant cytokine production (TNF-α, CCL1 and IL-13), whereas the activation of LIF was confined to SCF. SCF favored KIT, ERK, AKT, and STAT5 activation, whereas IL-33 preferentially stimulated JNK and p38 pathways. Although post-mitotic MCs showed diminished overall responsiveness to SCF, and with interesting differences among modules, their cytokine response to SCF remained comparable. Intriguingly, after exiting the cell cycle, MCs showed heightened sensitivity to IL-33, evidenced by increased ERK activation and TNF-α production. Collectively, IL-33 and SCF elicit markedly different early responses in human skin MCs. Chronic exposure to SCF reduces the responsiveness to this GF without eliminating their reactivity, while non-dividing MCs become more sensitive to IL-33, possibly as a compensatory adaptation.

## 1. Introduction

Mast cells (MCs) are tissue-resident immune cells and the major effector cells of IgE-mediated type-I-hypersensitivity encompassing allergic rhinoconjunctivitis, atopic asthma, food allergy and anaphylaxis [1,2]. In the skin, MCs are involved in various chronic dermatoses like urticaria, atopic eczema, contact dermatitis, psoriasis, prurigo and rosacea; conditions in which these cells are overabundant and/or hyperactive [3,4,5,6,7,8,9,10,11,12,13,14].

The SCF-receptor (KIT) represents the most significant receptor tyrosine kinase of the lineage implicated in differentiation, proliferation, survival and various functional aspects [15,16,17,18,19]. In skin MCs, the KIT ligand stem cell factor (SCF) triggers an intricate network of signaling cascades comprising changes in ≈5400 phosphosites [20]. The dominant pathways were found to be RAS/MEK (Mitogen-activated protein kinase)/ERK (Extracellular signal-regulated kinase), followed by PI3K (Phosphoinositide-3-kinase)/AKT (Protein kinase B) and STAT5 (Signal transducer and activator of transcription 5), while p38 and c-Jun N-terminal kinase (JNK) were weak or below detection [20]. In addition to survival promotion, SCF can also trigger cytokines, including the recently identified oncostatin M (OSM) and leukemia inhibitory factor (LIF) which belong to the interleukin-(IL-)6 family [20]. SCF-elicited cytokines typically require the action of ERK1/2 downstream of KIT.

IL-33 is another cytokine tightly linked to MC biology [21,22,23,24,25,26,27]. As a member of the IL-1 superfamily, it acts as an alarmin and pro-inflammatory mediator after being released into the extracellular space, e.g., from endothelial and epithelial cells during infection or sterile perturbation [28,29,30,31]. IL-33 is critical in the induction and maintenance of allergic disorders, such as asthma, allergic rhinitis, and skin disorders [32,33,34,35,36,37,38]. Skin MCs express the IL-33 receptor ST2 (gene name *IL1RL1*) at high levels and respond vigorously to IL-33 [39,40,41,42,43]. In contrast to SCF, IL-33 is unable to elicit cell cycle entry of mature skin MCs, yet it supports survival and is a very potent elicitor of cytokines [39,42]. Activation of three major cascades has been reported to occur downstream of ST2 in cutaneous MCs, namely p38, JNK and nuclear factor kappa-light-chain-enhancer of activated B cells (NF-κB) with minimal involvement of ERK or PI3K/AKT [39,40,41]. This is in line with p38 and NF-κB being the major modules orchestrating cytokine production downstream of ST2 [39,41].

Though SCF and IL-33 can both activate cytokine production, the quantitative and qualitative differences are ill-defined, as a direct comparison between the two stimuli has, to our knowledge, not been conducted in cutaneous MCs. Moreover, it is unclear whether IL-33 can elicit IEGs (immediate-early genes, e.g., *FOS* [Fos proto-oncogene], *JUN* [Jun proto-oncogene], *EGR1* [early-growth response 1]) in skin MCs, which can further contribute to cytokine generation.

Contrary to other myeloid cells (e.g., neutrophils, basophils and monocytes), which diminish their proliferative potential as they differentiate along their specific pathways from hematopoietic stem cells, MCs retain impressive mitogenic competence even as fully differentiated cells [7,15,19]. As would be expected for non-transformed cells, MCs proliferate for a limited period of time before they permanently exit the cell cycle and these post-mitotic MCs can be viewed as a model of aged MCs [44]. Post-mitotic cells still survive for a few weeks with their numbers declining gradually [44]. Since the cessation of proliferation occurs despite the constant presence of growth factors, it is unclear whether reduced sensitivity to SCF is involved in this switch. It is likewise unknown how post-mitotic MCs respond to other stimuli such as IL-33.

MC cytokines are critically implicated in chronic skin disorders and other diseases. In the current study, we therefore embarked on an investigation of the cytokine responses of skin MCs elicited by SCF vis-à-vis IL-33 in the cycling and post-mitotic phase. We report that IL-33 is typically a more potent elicitor of cytokines than SCF, but with interesting exceptions. In accordance, IEGs, which can contribute to cytokine responses, are also differentially regulated by IL-33 vis-à-vis SCF. Major distinctions are found for the preferential signaling pathways with complementary cascades being activated. After definite exit from the cell cycle, skin MCs still respond to SCF, though less potently and with adjustments in signaling modes. On the other hand, post-mitotic MCs retain or further upregulate sensitivity to IL-33. Post-mitotic MCs may be a major subset in the skin of the elderly population, and the mechanistic modifications that occur in MCs between the proliferative and the post-mitotic phase are therefore of substantial pathophysiologic and clinical relevance.

## 2. Materials and Methods

### 2.1. Cells and Treatments

MCs were isolated from human foreskin tissue as described [41]. Each MC preparation/culture originated from several (2–18) donors to achieve sufficient cell numbers, as is routinely performed in our lab [45,46,47]; the donor ages ranged from ≈2 to 18 years.

The skin was obtained from circumcisions, with written informed consent of the patients or their legal guardians, and approval by the university ethics committee (protocol code EA1/204/10, 9 March 2018). The experiments were conducted according to the Declaration of Helsinki principles and approved by the Institutional Ethics Committee of Charité–Universitätsmedizin Berlin (protocol code EA1/204/10, date 9 March 2018). Informed consent was obtained from all subjects or their legal guardians involved in the study. Briefly, the skin was cut into strips and treated with dispase (26.5 mL per preparation, activity: 3.5 U/mL; BD Biosciences, Heidelberg, Germany) at 4 °C overnight. The epidermis was removed, and the dermis was finely chopped and digested with 1.5 mg/mL collagenase (Worthington, Lakewood, NJ, USA), 0.75 mg/mL hyaluronidase (Sigma, Steinheim, Germany), and DNase I at 10 µg/mL (Roche, Basel, Switzerland) at 37 °C in a shaking water bath for 75 min. Cells were filtered stepwise from the resulting suspension (100 and 40 µm strainers, Fisher Scientific, Berlin, Germany). MC purification was achieved using anti-human c-Kit microbeads (#130-091-332) and an Auto-MACS separation device (both from Miltenyi-Biotec, Bergisch Gladbach, Germany), resulting in 98–100% pure preparations (using acidic toluidine blue staining, 0.1% in 0.5 N HCl (Fisher Scientific) as described [45].

Purified skin MCs from individual preparations were cultured in Basal Iscove’s medium with 10% FCS (both from Biochrom, Berlin, Germany) in the presence of SCF (100 ng/mL), and IL-4 (20 ng/mL) freshly provided twice weekly when the cultures were readjusted to 5 × 10^5^/mL. MCs were automatically counted using CASY-TTC (Innovatis/Casy Technology, Reutlingen, Germany). Post-mitotic MCs were defined as cells after long-term culture (minimum 8 weeks), for which a reduction in cell number was noted for two consecutive weeks. These cells are unable to incorporate BrdU anymore, as published previously [44]. Proliferating cells were defined as those cultured for 2–4 weeks, during which they exhibit maximal proliferative capability. Paired samples of proliferating and post-mitotic MCs (derived from different donor pools) were harvested and analyzed on the same day under identical conditions.

Experiments were performed 3–4 d after the last addition of cytokines. Cells were incubated in minimal medium without growth factors (GFs) and fetal calf serum (FCS) for at least 2 h, and were then stimulated with SCF or IL-33 for downstream experiments. Each experiment was performed on several individual cultures (given as a “dot” in the dot plots displayed in the figures). IL-33 was purchased from PeproTech (Hamburg, Germany) and applied at a concentration of 20 ng/mL, as described [40].

### 2.2. Reverse Transcription-Quantitative PCR (RT-qPCR)

Cells were stimulated with SCF (100 ng/mL) or IL-33 (20 ng/mL) for 30 min or 90 min and harvested for RNA extraction. The 30 min stimulation was used to assess the expression of immediate-early genes (IEGs), while stimulation for 90 min was used to examine cytokine expression profiles. RNA was isolated using the NucleoSpin RNA kit (Macherey-Nagel, Düren, Germany) following the manufacturer’s instructions. cDNA synthesis (reverse transcription kit from Fisher Scientific) and RT-qPCR were performed using the QuantiTect SYBR Green PCR Kit (QIAGEN, Hilden, Germany) under optimized cycling conditions, following the manufacturer’s protocol. The primer pairs are summarized in Table 1. They were synthesized by (TibMolBiol, Berlin, Germany). The 2^−ΔΔCT^ method was used to quantify the relative expression levels of the target genes to three reference genes (appearing at the end of Table 1).

### 2.3. Immunoblot Analysis

After stimulation with SCF (100 ng/mL) or IL-33 (20 ng/mL) for the indicated times, MCs were collected by centrifugation and immediately solubilized in SDS-PAGE (Sodium Dodecyl Sulphate-Polyacrylamide Gel Electrophoresis) sample buffer (Bio-Rad, Munich, Germany, cat-no. 1610737) and boiled for 15 min (whole-cell lysates). Samples with equal cell numbers were subjected to immunoblot analysis. Membrane blocking was performed in a 5% (*w*/*v*) low-fat milk powder (Carl Roth, Karlsruhe, Germany) solution for 30 min. The following primary antibodies were purchased from Cell Signaling Technologies (Frankfurt am Main, Germany): anti-pp38 (T180/Y182, #9211), anti-pSAPK/JNK (T183/Y185, #9251),anti-p-STAT5 (Y694, #9359), anti-pERK1/2 (T202/Y204, #9101), anti-pAKT (S473, #9271), anti-c-KIT (#3074), anti-p-c-KIT (Tyr719, #3391), anti-α-actinin (#6487) and anti-Cyclophilin B (#43603), the latter two serving as loading controls. For the detection antibody, a goat anti-rabbit IgG peroxidase-conjugated antibody was used (Merck, Darmstadt, Germany #AP132P). For the consecutive detection of several proteins on the same membrane, the antibodies (primary and secondary) were removed from the membrane after each detection step with incubation in 0.5 N NaOH (Carl Roth, Karlsruhe, Germany) for 15 min. After each stripping step, the membrane was blocked in 5% (*w*/*v*) low-fat milk powder for 30 min (as above), followed by incubation with the next primary antibody. Proteins were visualized using a chemiluminescence assay (Weststar Ultra 2.0, Cyanagen, Bologna, Italy) according to the manufacturer’s instructions. Bands were recorded on a chemiluminescence imager (Fusion FX7 Spectra, Vilber Lourmat, Eberhardzell, Germany). Semi-quantification of recorded signals was performed using ImageJ software (version 1.54g, Rasband, W.S., ImageJ, U. S. National Institutes of Health, Bethesda, MD, USA, https://imagej.net/ij/ (last accessed on 21 September 2025)). Individual intensity values for the detected proteins were normalized to the intensity of the housekeeping proteins cyclophilin B and α-actinin on the same membrane.

### 2.4. ELISA

Cells were stimulated with SCF (100 ng/mL) and IL-33 (20 ng/mL) for 24 h, and supernatants were collected. ELISA assays were performed to quantify cytokine levels using the following kits, according to the manufacturers’ instructions: Human LIF ELISA Kit (Cat# BMS242, Thermo Fisher Scientific, Berlin, Germany), Human IL-13 DuoSet ELISA (Cat# DY213, R&D Systems, Wiesbaden, Germany), Human CCL1/I-309 DuoSet ELISA (Cat# DY272, R&D Systems), and Human TNF-α ELISA Kit (Cat# 88-7346-22, Thermo Fisher Scientific).

### 2.5. Flow Cytometry

Mast cells were washed twice in Ca^2+^/Mg^2+^ free PBS, incubated with Zombie Violet (1:300, Biolegend, Koblenz, Germany) in PBS for 15 min and then blocked for 15 min at 4 °C with human AB-serum (Biotest, Dreieich, Germany) in FACS buffer (0.1% BSA, 0.5 mM EDTA). Thereafter, the cells were incubated with an anti-KIT-APC antibody (mAB: clone REA787 Miltenyi Biotec) for 30 min at 4 °C according to the manufacturer’s instructions. After one wash, cells were analyzed by a Sony ID7000 spectral analyzer (Sony Biotechnology, Berlin, Germany). For negative controls, cells were left unstained and stained with the corresponding isotype control antibody according to the manufacturer’s instructions.

### 2.6. Statistics

Statistical analysis was performed using GraphPad Prism software (version 10.4.2, GraphPad Software, San Diego, CA, USA). Comparisons between two groups were performed using the paired Student’s *t*-test. For comparisons across more than two groups, an ordinary one-way ANOVA with Dunnett’s multiple comparisons test was used. When comparing the stimulation groups with the control, one-sample *t*-tests were performed against the control value (1). A *p*-value of less than 0.05 was considered statistically significant.

## 3. Results

### 3.1. SCF and IL-33 Elicit Distinct Profiles of Immediate-Early Genes in Skin MCs

We previously demonstrated that SCF potently induces several IEGs in human skin MCs, including *NR4A2*, *EGR1*, and *FOS* [20,47,48]. Their induction was entirely ERK-dependent but PI3K-independent [20] and required the transcription factor CREB [48]. In contrast to the above genes, *JUN* (another early response gene) was only modestly induced or remained unaffected by most stimuli (unpublished observations). The regulation of IEGs by IL-33 in skin MCs has remained unexplored.

**Figure 1:**
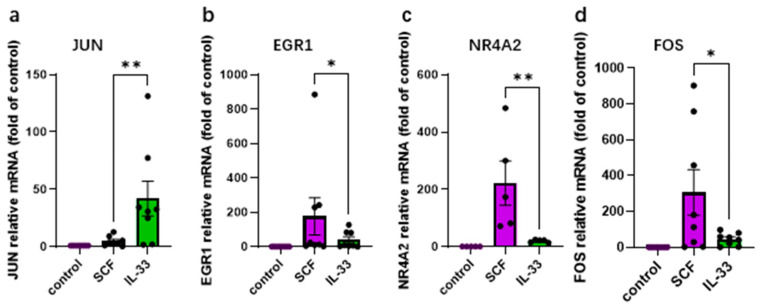
SCF and IL-33 elicit distinct IEG profiles in human skin MCs. Cells were stimulated with SCF (100 ng/mL) or IL-33 (20 ng/mL) for 30 min; RT-qPCR was used to quantify gene expression (normalized to housekeeping genes, as described in Methods). Results are expressed relative to the unstimulated control set as 1 and given as mean ± SEM and individual dots (each dot corresponding to a different skin MC culture). (**a**) *JUN*, (**b**) *EGR1*, (**c**) *NR4A2*, (**d**) *FOS*. * *p* < 0.05; ** *p* < 0.01. The figures were created using GraphPad Prism 10.4.2 (GraphPad Software, La Jolla, CA, USA, accessed on 23 February 2026).

In the current study, SCF and IL-33 both significantly upregulated all IEGs tested but elicited distinct levels of induction (Figure S1). While *EGR1*, *NR4A2*, and *FOS* were strongly upregulated with SCF, *JUN* was modestly affected by the growth factor (Figure 1a–d and Figure S1). Conversely, IL-33 elicited pronounced upregulation of *JUN* (Figure 1a and Figure S1). Therefore, a complementary pattern became apparent between the growth factors, suggesting stimulus-specific engagement of transcriptional programs. IL-33 may preferentially trigger *JUN* expression via JNK activation, while SCF may induce ERK-dependent IEGs in the first place.

### 3.2. SCF and IL-33 Elicit Distinct Cytokine Profiles, with IL-33 Being Overall More Potent

Cytokines elicited by IL-33 and SCF were independently assessed in previous studies but not assessed side-by-side in skin MCs [20,41]. We therefore examined the activation of typical cytokines by the growth factors under the otherwise identical experimental conditions.

**Figure 2:**
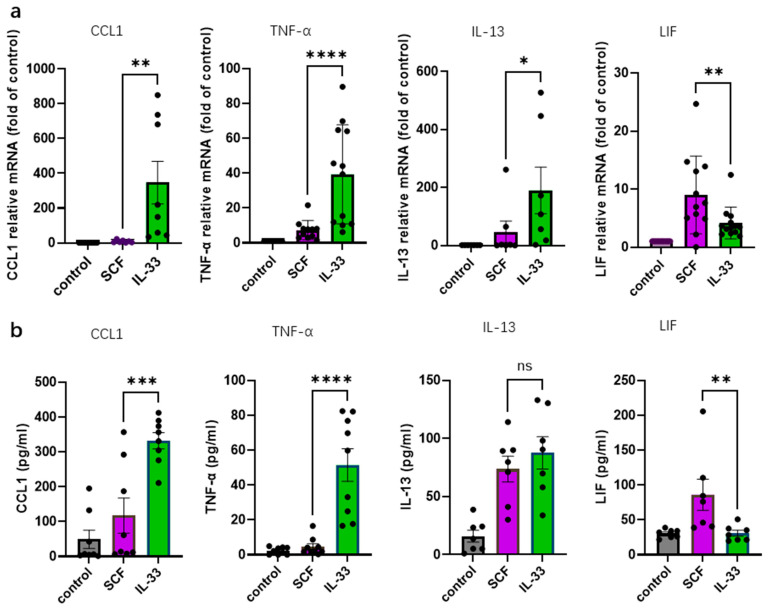
IL-33 and SCF differentially modulate cytokine production in skin MCs. (**a**) mRNA levels of *CCL1*, *TNF-α*, *IL-13*, and *LIF* were assessed by RT-qPCR following stimulation with IL-33 (green) or SCF (purple) for 90 min. Expression was normalized to three housekeeping genes (*ACTB*, *GAPDH*, and *PPIB*) and is presented relative to the unstimulated control. (**b**) Protein concentrations of CCL1, TNF-α, IL-13, and LIF were determined by ELISA in culture supernatants after stimulation. The data are presented as individual dots with mean ± SEM. * *p* < 0.05; ** *p* < 0.01; *** *p* < 0.001; **** *p* < 0.0001; ns: not significant. The figures were created using GraphPad Prism 10.4.2 (GraphPad Software, La Jolla, CA, USA, accessed on 23 February 2026).

Figure 2a shows the FC (fold change) over control at the mRNA level, revealing that IL-33 robustly induced *TNF-α*, *CCL1*, and *IL-13* transcripts which are key pro-inflammatory or Th2-associated cytokines, respectively (Figure 2a). This also applied to *CXCL8* which encodes IL-8, and *CCL2* (Figure S2). On the other hand, SCF had minimal effect on *CCL1* and *CXCL8* transcripts, while eliciting some *TNF-α* and *IL-13,* though less efficiently than IL-33 (Figure 2a and Figure S1).

Conversely, *LIF* mRNA was preferentially induced by SCF over IL-33 (Figure 2a). OSM, another IL-6 family cytokine, was also primarily stimulated by SCF (Figure S1).

We also examined the protein levels of selected cytokines. CCL1, TNF-α, LIF, and IL-13 were chosen for validation due to their robust expression in skin MCs. IL-33 elicited significantly greater secretion of CCL1, and TNF-α than SCF (Figure 2b). Conversely, no difference in IL-13 secretion was noted. SCF induced markedly higher LIF production than IL-33 in accord with the mRNA data. LIF is produced and released by skin MCs also without stimulation, but basal levels can be further enhanced by SCF [20]. We now show that, in contrast to SCF, IL-33 is unable to elevate LIF above baseline, suggesting different prerequisites underlying its stimulation.

### 3.3. Differential Activation of Signaling Pathways by SCF and IL-33: SCF Is Dominant at p-KIT and p-ERK1/2, While IL-33 Preferentially Activates JNK and p38

SCF and IL-33 induce distinct signaling cascades in skin MCs [20,39], but no kinetic resolution is available for the growth factors examined simultaneously (i.e., on the same blots).

**Figure 3:**
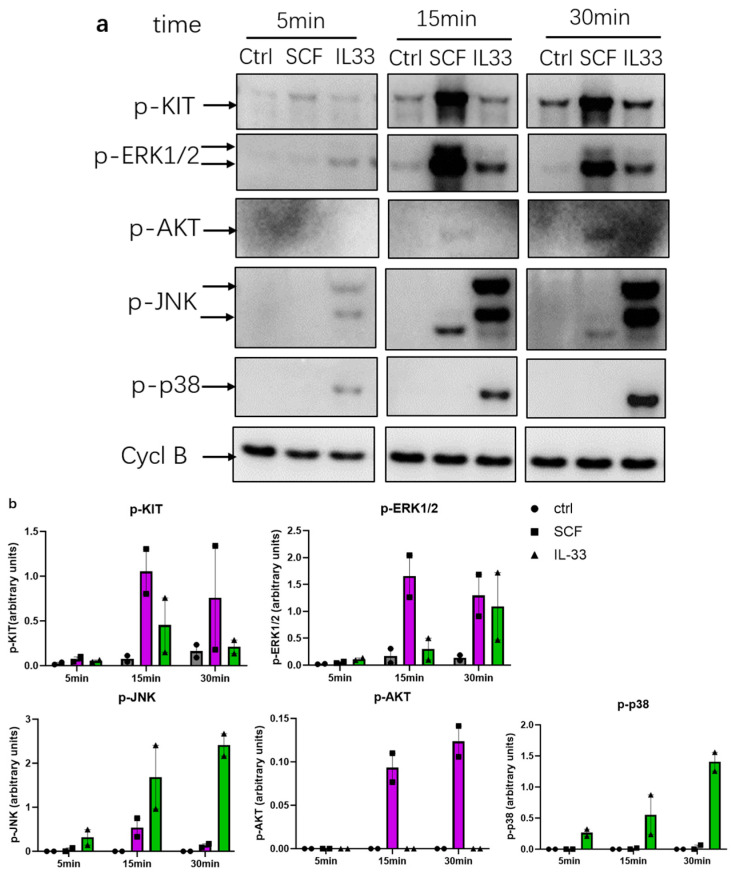
Signaling pathways differ strongly between SCF and IL-33. Skin MCs were stimulated with IL-33 (20 ng/mL) or SCF (100 ng/mL) for the indicated times, and phosphorylation of KIT, ERK1/2, JNK, p38, and AKT were detected by immunoblotting. Phosphorylated ERK1/2 and JNK appear as double bands corresponding to different isoforms. Cyclophilin B (Cycl B) served as the loading control. Notably, a smaller band observed in the SCF condition for p-JNK is likely non-specific, as it differs in size from the expected JNK isoforms detected upon IL-33 stimulation. Weak pAKT activation is only visible after SCF stimulation. Ctrl, cells treated with vehicle control. Representative of 2 independent blots. (**b**) Densitometric analysis of two independent immunoblots (including the one shown in (**a**)) was performed using ImageJ. Phospho-protein signals were normalized to Cyclophilin B. The data are presented as mean ± SEM. The figures were created using GraphPad Prism 10.4.2 (GraphPad Software, La Jolla, CA, USA, accessed on 23 February 2026).

Comparing the two, we found that stimulation with SCF results in pronounced and consistent phosphorylation of KIT and ERK1/2 at 15 and 30 min (Figure 3a,b), aligning with the well-documented KIT-mediated activation of this MAPK [20,46,48]. In contrast, IL-33 (20 ng/mL) induced barely any p-KIT and only modest p-ERK1/2, in line with previous observations [42]. Along the same lines, p-AKT was slightly induced by SCF, but not by IL-33.

Conversely, IL-33 induced robust phosphorylation of JNK and p38, which became evident at 5 min but steadily increased till 30 min (Figure 3a,b). In contrast, the activation of JNK or p38 was below detection with SCF.

### 3.4. Selective Modules of SCF-Induced Signaling Are Attenuated in Post-Mitotic Compared to Proliferative MCs

After a predetermined number of duplications, skin MCs definitely exit the cell cycle [44]. We asked whether this is partially caused by altered SCF sensitivity.

**Figure 4:**
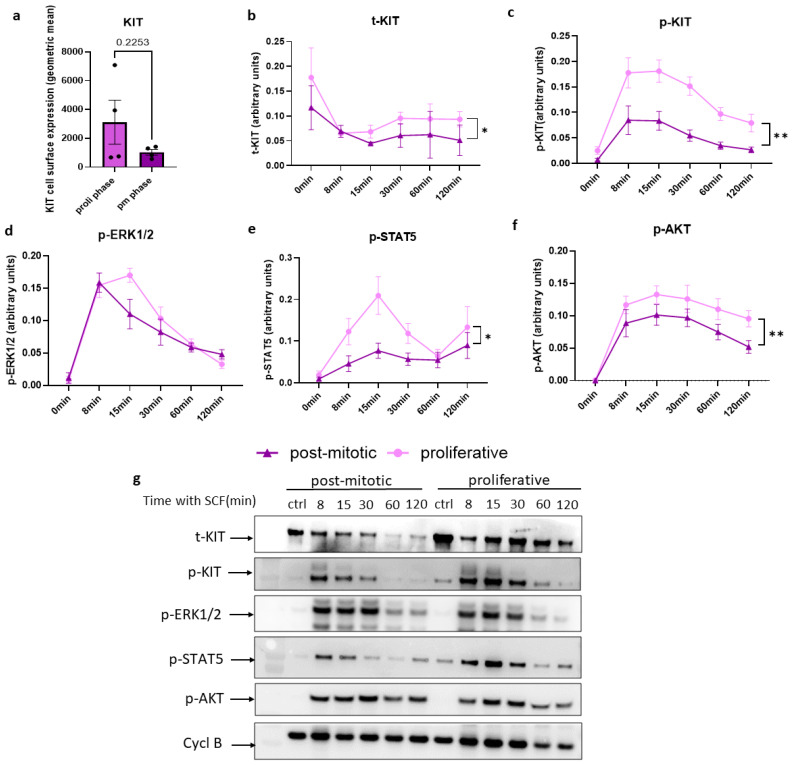
Post-mitotic MCs are overall less responsive to SCF. (**a**) Cells corresponding to the two phases were analyzed by flow cytometry for KIT surface expression. The data are presented as individual dots with mean ± SEM. pm indicates post-mitotic MCs; proli indicates proliferative MCs. (**b**–**g**): MCs were stimulated with SCF for the indicated periods, harvested, and subjected to Western blot analysis, using the indicated antibodies. An antibody against Cyclophilin B (Cycl B) was included as the loading control (shown in (**g**)) and was used for normalization (**b**–**f**). (**b**–**f**) Relative quantifications of the Western blot signals. The means ± SEM of four independent experiments are shown. * *p* < 0.05; ** *p* < 0.01 by paired *t*-test of the AUC (area under the curve). (**g**) Representative Western blot experiment, using the indicated antibodies. The membrane was sequentially incubated with antibodies against p-KIT and p-ERK1/2, followed by p-AKT and Cyclophilin B. After stripping, the membrane was re-probed with anti-p-STAT5 antibody. Subsequently, after a second stripping, the membrane was incubated with anti-total KIT antibody. The order of the membranes shown here matches the statistical analyses above (**b**–**f**). p- and t-indicate phosphorylated and total forms of the proteins. The figures (**a**–**f**) were created using GraphPad Prism 10.4.2 (GraphPad Software, La Jolla, CA, USA, accessed on 23 February 2026).

To test this, MCs, unfed for 3–4 days and deprived of SCF for 2 h, were washed and stimulated with the growth factor for different times. To ensure comparability, viability was first assessed by flow cytometry using Zombie Violet staining, confirming similarly high survival in both proliferative and post-mitotic MCs (Figure S3). The total KIT expression trended to be higher in proliferative MCs by Western blot and by flow cytometry without reaching significance (Figure 4a,b). SCF led to rapid reduction in KIT through internalization and degradation [49]. This reduction was comparable between post-mitotic and proliferative MCs (Figure 4b).

Phosphorylated KIT (Y719) levels peaked at 8–15 min in both groups but were significantly higher and more sustained in proliferative MCs. In contrast, post-mitotic cells showed blunted and transient activation, indicating reduced KIT signaling capacity (Figure 4b). As downstream effectors, ERK1/2 and STAT5 were both activated following SCF stimulation (Figure 4c,d). Post-mitotic MCs exhibited little difference in ERK phosphorylation against proliferating MCs, while the phosphorylation of STAT5 was clearly reduced in non-cycling cells (Figure 4c,d). The phosphorylation of AKT was similar to STAT5 and markedly elevated in actively cycling cells (Figure 4e). Together, the data indicate that post-mitotic MCs exhibit attenuated but not eliminated activation of KIT-dependent pathways in response to SCF, and with some differences across downstream modules.

### 3.5. IL-33-Induced ERK Activation Increases in Post-Mitotic MCs

**Figure 5:**
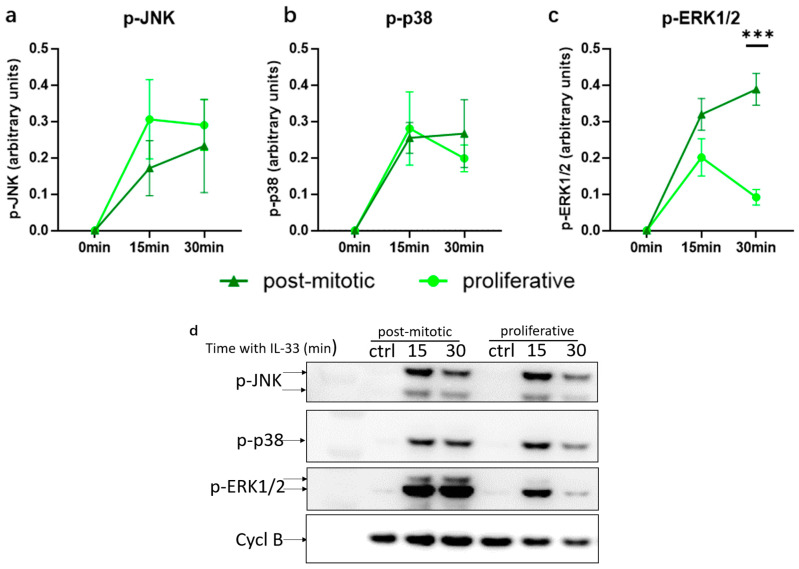
IL-33-induced ERK phosphorylation is enhanced in post-mitotic MCs. MCs were stimulated with IL-33 for the indicated periods. Cells were harvested and subjected to Western blot analysis using antibodies against p-ERK, p-JNK, and p-p38. An antibody against Cyclophilin B was included as a loading control and was used for normalization. (**a**–**c**) Relative quantifications of the Western blot signals. The means ± SEM of four independent experiments are shown. *** *p* < 0.001. (**d**) Representative Western blot experiment, using the indicated antibodies. The membrane was sequentially incubated with antibodies against p-p38, then stripped and re-probed with p-JNK, followed by Cyclophilin B and finally p-ERK1/2. The order shown here matches the statistical analyses above (**a**–**c**). p- indicates phosphorylated forms of the proteins. The figures (**a**–**c**) were created using GraphPad Prism 10.4.2 (GraphPad Software, La Jolla, CA, USA, accessed on 23 February 2026).

We queried whether the reduced signaling responses of post-mitotic MCs were SCF-specific or a more general phenomenon. We therefore assessed the degree of IL-33-induced p-ERK, p-JNK and p-p38 comparatively for proliferating versus post-mitotic MCs. JNK and p38 were robustly activated with no significant differences between subsets (Figure 5a,b). Surprisingly, however, there was a stronger and more persistent activation of ERK1/2 in the non-proliferating cells (Figure 5c). Overall, this suggests that post-mitotic MCs remain fully responsive to IL-33, even strengthening the ERK module that is only weakly activated in proliferating skin MCs.

### 3.6. Post-Mitotic MCs Show Enhanced IL-33-Induced Cytokine Responses, While Responses to SCF Are Broadly Similar to Those of Proliferative Cells

As shown in Figure 4 and Figure 5, SCF and IL-33 stimulation led to distinct activation patterns of key signaling pathways in proliferative and post-mitotic MCs. Specifically, SCF-induced signaling was attenuated in post-mitotic cells, while IL-33-induced ERK activation simultaneously increased. These findings prompted us to explore how these differences impact cytokine expression in the two cell states. mRNA expression and protein secretion of selected cytokines were measured after stimulation with SCF or IL-33 under identical conditions.

**Figure 6:**
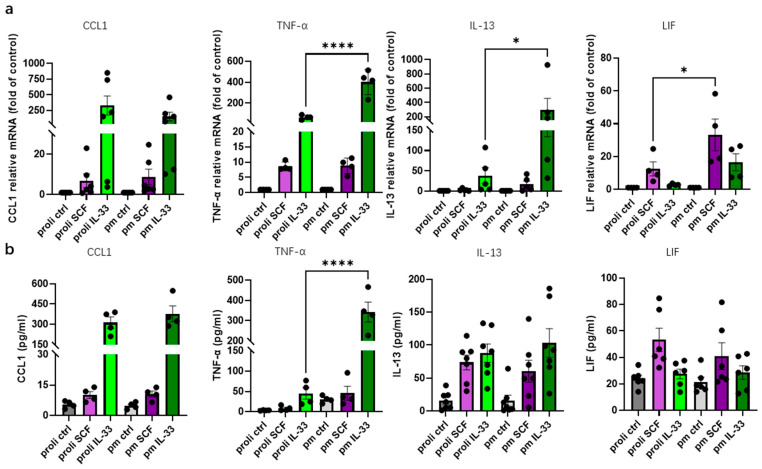
IL-33-induced cytokine responses tend to be more pronounced in post-mitotic cells, while SCF responsiveness remains broadly comparable between lifecycle phases. (**a**) mRNA levels of *CCL1*, *TNF-α*, *IL-13* and *LIF* were assessed by RT-qPCR in proliferative and post-mitotic MCs following stimulation with IL-33 (green) or SCF (purple) for 90 min. Expression was normalized as described in Methods and is presented relative to the unstimulated control. (**b**) Protein concentrations of CCL1, TNF-α, IL-13, and LIF were determined by ELISA in culture supernatants from proliferative and post-mitotic MCs. The data are presented as individual dots with mean ± SEM. * *p* < 0.05; **** *p* < 0.0001. pm indicates post-mitotic MCs; proli indicates proliferative MCs. The figures were created using GraphPad Prism 10.4.2 (GraphPad Software, La Jolla, CA, USA, accessed on 23 February 2026).

Figure 6a shows the FC (fold change) over control for mRNA expression. Under SCF stimulation, cytokine expression was largely comparable between proliferative and post-mitotic stages, except for a significant difference in higher *LIF* expression in post-mitotic cells (Figure 6a). LIF secretion remained unchanged between the cell states at the protein level though (Figure 6b).

IL-33 stimulation led to more pronounced differences between stages. Notably, stimulated *TNF-α* and *IL-13* levels were significantly elevated in post-mitotic cells at the transcriptional level. There was also a trend for LIF without reaching significance, while CCL1 expression did not differ between stages. While changes in *IL-13* (and *LIF*) were confined to the transcript level, IL-33-induced secretion of the TNF-α protein was also efficiently upregulated in non-cycling cells.

Together, these findings suggest that cytokine stimulability by SCF remains intact after MCs have exited the cell cycle. Conversely, cytokine responses elicited by IL-33 are either unchanged or enhanced in post-mitotic cells, with pronounced increments especially for TNF-α.

## 4. Discussion

SCF and IL-33 target two key receptors of the MC lineage. SCF dimerizes its receptor KIT, activating its intrinsic kinase activity and thereby orchestrating maturation, proliferation, survival, and function [15,16,17,18,19]. IL-33, a member of the IL-1 family, constitutes a potent alarmin released by damaged or infected tissue [31,50,51]. IL-33 signals via the ST2/IL1RacP (Interleukin-1 Receptor Accessory Protein) complex, and also forms a component of the MC-supportive micromilieu [22,52,53,54,55,56]. This is illustrated by the finding that ST2 deficiency is associated with reduced MCs numbers, while exogenous IL-33 can numerically strengthen the MC compartment, suggesting IL-33’s involvement in its long-term maintenance [57,58]. While IL-33 is not mitogenic to skin MCs on its own, it can enhance proliferative signals from SCF/KIT [42], whereas in other types of MCs, it may even elicit proliferation independently of KIT [58].

In the current study, we first confirm that signaling pathways differ profoundly between the SCF/KIT and IL-33/ST2 axes in skin MCs, extending our previous research [20,39,41,45,48]. We highlight that SCF primarily activates p-KIT, p-ERK, p-AKT, and p-STAT5, while IL-33 triggers p-JNK and p-p38, as well as the NF-κB pathway (the latter not studied here), emphasizing the distinct and complementary nature of the signaling machineries contracted by KIT and ST2. Similar, yet less clear-cut differences have been reported in murine MCs [59].

It is also of interest to compare the patterns to other crucial activatory receptors of the lineage, such as FcεRI and MRGPRX2, which trigger MC degranulation and the release of newly formed mediators, but, in contrast to ST2 and KIT, are not primarily associated with growth or survival; conversely ST2 and KIT barely elicit granule discharge. Notably, FcεRI and MRGPRX2 show greater resemblance to the SCF/KIT system by also eliciting robust ERK and AKT phosphorylation (albeit with different kinetics), combined with poor p-p38 and p-JNK responses [60]. It therefore seems that the IL-33/ST2 axis is quite unique, further substantiated by the fact that human skin MCs barely express Toll-like receptors or IL-1R1 [43,61], which share signaling pathways with ST2. Activation of the IL-33 receptor may therefore be critical to the rapid sensing of perturbations by MCs in the cutaneous microenvironment. It is also because of this difference in signaling cascades that IL-33 synergizes so potently with other receptors through complementation of modules [40,41].

IEGs are rapidly induced by various stimuli independently of new protein expression [62,63]. We recently reported that KIT, but also FcεRI, drives the induction of typical IEGs like *NR4A2*, *EGR1*, *JUNB*, *FOSB* and *FOS* [20,47,48,64]. IEGs often encode TFs that activate further response genes in a cascade-like fashion. In our previous study, IEG induction by SCF depended strictly on ERK, whereby ERK2 seemed to be the dominant isoform [20]. It was later found that ERK operated by activating CREB in the first place, which was essential for the activation of all entities studied (*NR4A2*, *JUNB*, *FOS*) [48]. JUN, the other extensively studied AP-1 subunit aside from FOS, was barely induced by SCF or any other stimulus in skin MCs, however (unpublished results).

We now show that IL-33 is a potent inducer specifically of *JUN*, while IL-33 drives limited expression of *NR4A2*, *EGR1* or *FOS* compared to SCF. Therefore, the distinct signaling modules activated by KIT versus ST2 have a correlate in the profiles of IEGs they preferentially elicit, with *JUN* induction being largely confined to IL-33. This provides further evidence that the signaling pathways activated by the GF systems are highly distinct.

The favored activation of *JUN* by IL-33 is likely the result of JNK pathway activation, consistent with the observation that JNK activation by SCF was modest at best [20,42], as confirmed in this study. To our knowledge, selective upregulation of *JUN* by IL-33 has not been reported so far in any cell type, in contrast to JUN phosphorylation [65,66,67,68]. However, our findings are in accord with previous observations showing that active JNK can also drive JUN expression in addition to its phosphorylation [69,70]. This seems to be the result of autoregulation of *JUN* through dominant AP-1 binding sites, as JUN promotes its own gene’s transcription, thereby creating feed-forward loops [71]. Hence, the AP-1 complexes that form after stimulation with SCF versus IL-33 may profoundly differ in composition, with IL-33 potentially creating JUN:JUN homodimers as well as FOS:JUN heterodimers, the latter major initiators of further processes owing to the activatory function of both subunits [71]. Of note, other JUN members (e.g., JUNB, JUND) are poor transactivators in FOS:JUN complexes [71].

Remarkably, a sizeable degree of variability was observed in the transcriptional and other responses of skin MCs to SCF and IL-33. This variability is largely attributable to donor-dependent heterogeneity, an inherent characteristic of primary human MCs that has been consistently documented [72,73]. Such heterogeneity is likely driven by donor-specific epigenetic landscapes, which shape MC responsiveness to external stimuli, including SCF and IL-33 [42]. A similar degree of variability has also been reported by other laboratories [74]. While factors such as donor age or developmental origin may contribute, our prior work demonstrated comparable variability within pediatric and adult cohorts with little difference between them [73], indicating that these parameters alone do not account for the observed differences. To reduce inter-individual variability, the current study employed pooled MCs from multiple donors. This strategy mitigates, but does not fully eliminate, inherent biological heterogeneity.

IEGs are well-known to feed into the generation of cytokines in MCs, since both AP-1 and EGR1 can bind to crucial elements within regulatory sequences of cytokine genes [75,76,77,78]. Indeed, MCs are believed to mainly act by soluble factors, and cytokines make up an important portion of their secretome. Since MCs localize near blood vessels at the interfaces that contact the outside world, they are strategically localized to act as primary responders organizing host defenses against intruding pathogens [79,80]. A substantial portion of these defense functions relies on MC-derived cytokines.

IL-33 has long been known as a potent activator of cytokines in various types of MCs [22,81,82,83,84,85,86,87,88,89], including human skin MCs [39,41]. SCF, on the other hand, can likewise stimulate cytokines in these cells [20], but how this compares to IL-33 is poorly defined.

We find herein that, on direct comparison, IL-33 is the more potent trigger of cytokines than SCF. This applies to CCL1, TNF-α, IL-8 and IL-13. Our data are in line with a previous report showing IL-33’s superiority at inducing TNF-α and IL-6 in murine MCs [59].

Some discrepancy between the RNA and protein level was detected for IL-13, whose transcript was more potently stimulated by IL-33, while the secreted protein was comparable for SCF and IL-33. In fact, not only IL-33 [81,83,84,86,90,91,92,93], but also SCF was previously described to elicit IL-13 responses in MCs [78,94]. The latter may be facilitated by the activation of STAT5, a transcription factor that reportedly contributes to IL-13 production [92]. The incongruent regulation of IL-13 RNA and protein on the other hand is compatible with previous observations: For example, the IL-13 transcript was found to be pre-formed in ex vivo skin MCs, while no protein could be detected [43,95]. In the current study, a disconnect was also observed for proliferating versus post-mitotic cells, whereby the latter responded to IL-33 stimulation with enhanced IL-13 mRNA induction, while IL-13 levels in the supernatants were unaltered. Possibly, the potency of IL-13 in Th2-type tissue destruction has led to the establishment of a further layer of regulation between transcription and translation/secretion. This is corroborated by previous results in primary human leukocytes for which a relatively poor correlation between IL-13 mRNA and protein was found (in comparison to other cytokines such as TNF-α) [96,97]. For CCL1 and TNF-α, we found a largely consistent upregulation at transcript and protein level in this study, while a previous report found greater synergy for TNF-α at protein compared to RNA regulation on simultaneous stimulation of distinct receptors [41]; therefore, the correlation between RNA and protein may also depend on the context in the case of TNF-α.

Interestingly, however, the induction of the IL-6 family members LIF and OSM was largely confined to SCF, as their levels after IL-33 stimulation were similar to baseline. OSM and especially LIF are also produced in MCs and constitutively secreted, yet they are further stimulated by SCF [20,43,98]. Conversely, IL-6 itself is unaffected by the stimulation of skin MCs (contrasting with other MC types, especially murine bone-marrow-derived cultured MCs [39,84,86,95,99]), and its expression is overall highly variable across skin donors [100].

LIF and OSM both require ERK activity for SCF-stimulated expression in skin MCs [20]. The reason for the virtual inability of IL-33 to upregulate LIF (in contrast to its greater potency at inducing other cytokines) may be the weaker activation of ERK by IL-33, leading to lower AP-1 activity through missing FOS or FOSB components. Of greater relevance even may be LIF’s dependence on STAT5 activity, with it being a direct target gene of STAT5 [101]. STAT5 is strongly activated by SCF [20], but not by IL-33 [42], as confirmed again in the current study, potentially explaining SCF’s potency at activating the LIF promoter. LIF (and OSM) have homeostatic functions in development and tissue regeneration, hematopoiesis, fertility, and the development of the nervous system [102,103].

Collectively, we document that SCF and IL-33 drive distinct cytokine profiles in skin MCs with regulatory cytokines like LIF being preferentially stimulated by SCF, while IL-33 elicits pro-inflammatory and Th2-type cytokines more potently. How the simultaneous stimulation with SCF and IL-33 might further influence these responses is another interesting question. Such experiments are currently underway, and preliminary data suggest that SCF and IL-33 may exert synergistic effects at least on certain cytokines. Further studies will be required to systematically investigate the nature, and extent of this phenomenon.

In their native tissues, MCs are long-lived and do not normally proliferate, but are capable of homeostatic proliferation, as elegantly shown in early studies [104,105]. Accordingly, injections of exogenous SCF in vivo have been demonstrated to cause substantial increments in MC numbers in mouse and man [106,107,108,109]. In vitro propagation of tissue MCs also requires substantial amounts of SCF. Ex vivo, skin MCs cycle for a limited period of time and survive for a few weeks thereafter, whereby the expression of PCNA (proliferating cell nuclear antigen) and incorporation of BrdU parallel their numerical changes [44]. These cells retain their phenotypic and functional properties, though they are unable to progress through the cell cycle despite high concentrations of SCF. Most importantly, they still respond to all stimuli tested, especially FcεRI aggregation, providing evidence that they are not “exhausted”; this is in contrast to what was reported for lymphocytes with their reduced effector functions and sustained expression of inhibitory receptors [104,105]. In addition to FcεRI aggregation [44], these nonproliferating MCs are also (still) partially responsive to SCF and fully responsive IL-33 (as shown in this study). Due to their terminal exit from the cell cycle, we refer to these cells as “post-mitotic”. In contrast, nonproliferating MCs can encompass also those that have not entered the cell cycle (yet) (which applies to most tissue MCs in vivo) [44].

Because of their rather short existence in vitro, the biology of post-mitotic MCs has barely been addressed, though such cells can serve as a model of aged MCs. Clearly, mitogenesis is a dramatic event in a cell’s existence, and cells undergoing replication can differ from their non-cycling counterparts [105,110]. We now find that growth and post-growth MCs do respond distinctly to SCF and IL-33. In fact, on chronic exposure to SCF, MCs become less responsive to the growth factor, but do not lose reactivity completely. Notably, KIT also responds differently in post-mitotic cells, since especially the Tyr719-KIT/PI3K/AKT and STAT5 modules are affected, while ERK seems barely perturbed vis-à-vis growing cells.

Interestingly, this reduced stimulability of MCs after exit from the cell cycle is limited to SCF, since cells simultaneously retain (or even further upregulate) sensitivity to IL-33, displaying greater ERK phosphorylation and increased cytokine responses. Therefore, post-mitotic cells are not characterized by general exhaustion and unresponsiveness to other growth-related stimuli.

The stronger ERK activation by IL-33 in post-mitotic MCs may stem from a greater interaction between c-Kit, IL-1RAcP and ST2, a complex described for other types of MCs [89]. In fact, while p38 is innate to the IL-33/ST2 cascade, ERK seems to require co-activation by KIT [89]. From a biological standpoint, post-mitotic cells may upregulate alternative GF networks simultaneously while reducing sensitivity towards SCF, and the enhanced stimulability by IL-33 may thus play a compensatory role. Since ERK is deeply interwoven in the expression of TNF-α and other cytokines in skin MCs [20,41], the enhanced activation of ERK by IL-33 in post-mitotic cells likely contributes to the cells’ parallel increase in cytokinergic potential.

## 5. Conclusions

This study established that the two major axes, SCF/KIT and IL-33/ST2, explored side by side in this study, differentially stimulate signaling pathways, immediate-early genes, and functional programs in human skin MCs. In this regard, SCF/KIT preferentially triggers the activation of ERK, PI3K/AKT, and STAT5, complemented by several IEGs (*EGR1*, *NR4A2*, *FOS*) and cytokines (especially LIF). The IL-33/ST2 axis, in contrast, potently activates p38 and JNK (but little ERK) and selectively drives the expression of the IEG *JUN*. IL-33 also induces a richer panel of cytokines than SCF (CCL1, TNF-α, IL-13). This reveals their complementary nature.

When optimally supported by high levels of SCF, MCs enter the cell cycle and grow for a limited time in culture before definitely exiting the cell cycle. Such post-mitotic MCs can serve as a model of aged MCs but have been poorly explored. We uncover molecular underpinnings differentiating proliferating from post-mitotic skin MCs. While the latter become less responsive to SCF, not all signaling modules are affected to the same extent. Strongly decreased are PI3K/AKT and STAT5, while ERK remains relatively stable, suggesting quantitative differences in signaling outputs downstream of KIT between these MC subsets.

In contrast to the diminished responsiveness to SCF, post-mitotic MCs enhance sensitivity to IL-33. This is detectable at the level of signaling pathways (increased ERK activation) as well as cytokine induction, especially of TNF-α. We assume that cells after exit from the cell cycle lower certain modules (such as Tyr719-KIT/PI3K or STAT5), while ERK, JNK, or p38 remain stable. This may result in halted proliferation combined with unperturbed or even increased cytokine outputs. The overall reduction in KIT signaling may favor shifts towards alternative factors such as IL-33.

## Figures and Tables

**Table 1:** Primer pairs used for RT-PCR.

Gene	Forward 5′-3′	Reverse 5′-3′
*TNF-α*	TCTCGAACCCCGAGTGACAA	TCAGCCACTGGAGCTGCC
*CXCL8*	ATGACTTCCAAGCTGGCCGTGGCT	TCTCAGCCCTCTTCAAAAACTTCTC
*CCL1*	TTGCGGAGCAAGAGATTCCC	GGCAGTGCCTCAGCATTTTT
*CCL2*	CCCCAAGCAGAAGTGGGTTC	TTGGGTTGTGGAGTGAGTGTT
*IL-13*	CATCCGCTCCTCAATCCTCT	GATGCTCCATACCATGCTGC
*LIF*	GAACCTCTGAAAACTGCCGG	TTGGCTCCTGATCTGGTTCA
*OSM*	GAGACTCATGACCAGGGGAC	CCCAGCTCCCACCTCTTAAA
*JUN*	CTGCCACCAATTCCTGCTTT	TTTCAGGAGGCTGGAGGAGG
*EGR1*	CTTCCCTTCCTCAGCTGTCA	TAGAGAGGGAGGACTTGGCT
*NR4A2*	TTCTGTAACCCTCCTAGCCC	AGCATGGCCAAACATTTCCC
*FOS*	AGTGACCGTGGGAATGAAGT	GCTTCAACGCAGACTACGAG
*ACTB*	CTGGAACGGTGAAGGTGACA	AAGGGACTTCCTGTAACAATGCA
PPIB *	AAGATGTCCCTGTGCCCTAC	ATGGCAAGCATGTGGTGTTT
*GAPDH*	ATCTCGCTCCTGGAAGATGG	AGGTCGGAGTCAACGGATTT

* The *PPIB* gene encodes Cyclophilin B.

## Data Availability

The original contributions presented in this study are included in the article/Supplementary Materials. Further inquiries can be directed to the corresponding author(s).

## Supplementary Materials

The following supporting information can be downloaded at https://www.mdpi.com/article/10.3390/cells15050398/s1, Figure S1: SCF and IL-33 Induce IEG transcription in human skin MCs; Figure S2: IL-33 and SCF differentially regulate cytokine production in skin MCs; Figure S3: Proliferating and post-mitotic MCs are characterized by high viability.

## Abbreviations

The following abbreviations are used in this manuscript:

AKT	Protein kinase B
EGR1	Early growth response 1
ERK	Extracellular signal-regulated kinase
FOS	Fos proto-oncogene
IEGs	Immediate-early genes
IL-33	Interleukin 33
JNK	c-Jun N-terminal kinase
JUN	JUN proto-oncogene
KIT	SCF-receptor
LIF	Leukemia inhibitory factor
MEK	MAPK kinase
NF-κB	Nuclear factor kappa-light-chain-enhancer of activated B cells
OSM	Oncostatin M
PI3K	Phosphoinositide-3-kinase
SCF	Stem cell factor
STAT5	Signal transducer and activator of transcription 5
TNF-α	Tumor necrosis factor alpha
