# Supplementary material for: Differential Responsiveness of Human Skin Mast Cells to SCF and IL-33: Reduced Reactivity to SCF but Not to IL-33 in the Post-Mitotic Phase
Source: Cells. 2026 Feb 24;15(5):398. doi: 10.3390/cells15050398 (PMC12984688; doi:10.3390/cells15050398)
Supplement: Supplementary file 1 [file cells-15-00398-s001.zip › cells-4021759-supplementary.pdf]

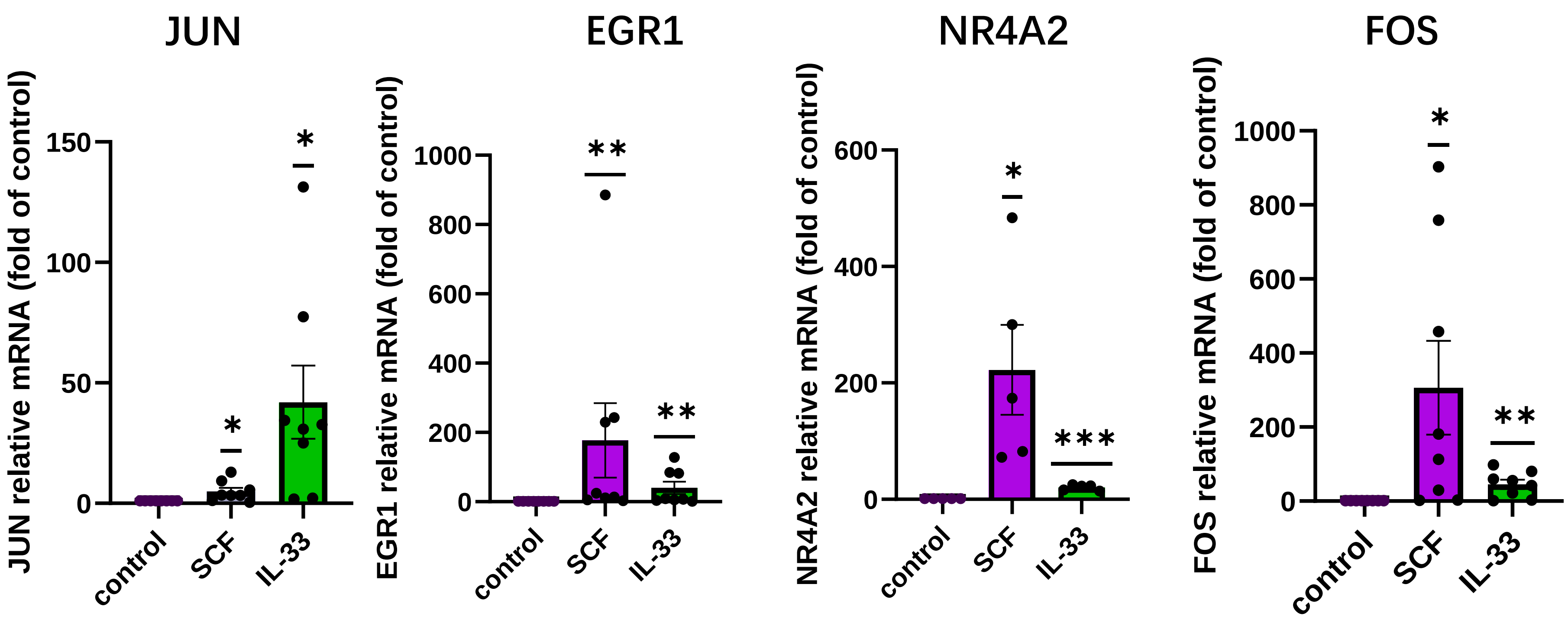

Figure S1: **SCF and IL-33 Induce IEG transcription in human skin MCs.** mRNA levels of *JUN*, *EGR1*, *NR4A2*, and *FOS* were assessed by RT-qPCR following stimulation with IL-33 (green) or SCF (purple) for 30 minutes. Expression was normalized to three housekeeping genes (*ACTB*, *GAPDH*, and *PPIB*) and is presented relative to the unstimulated control set as 1. The data are presented as individual dots with mean ± SEM. *p < 0.05; **p < 0.01; ***p < 0.001 by one-sample t-test.

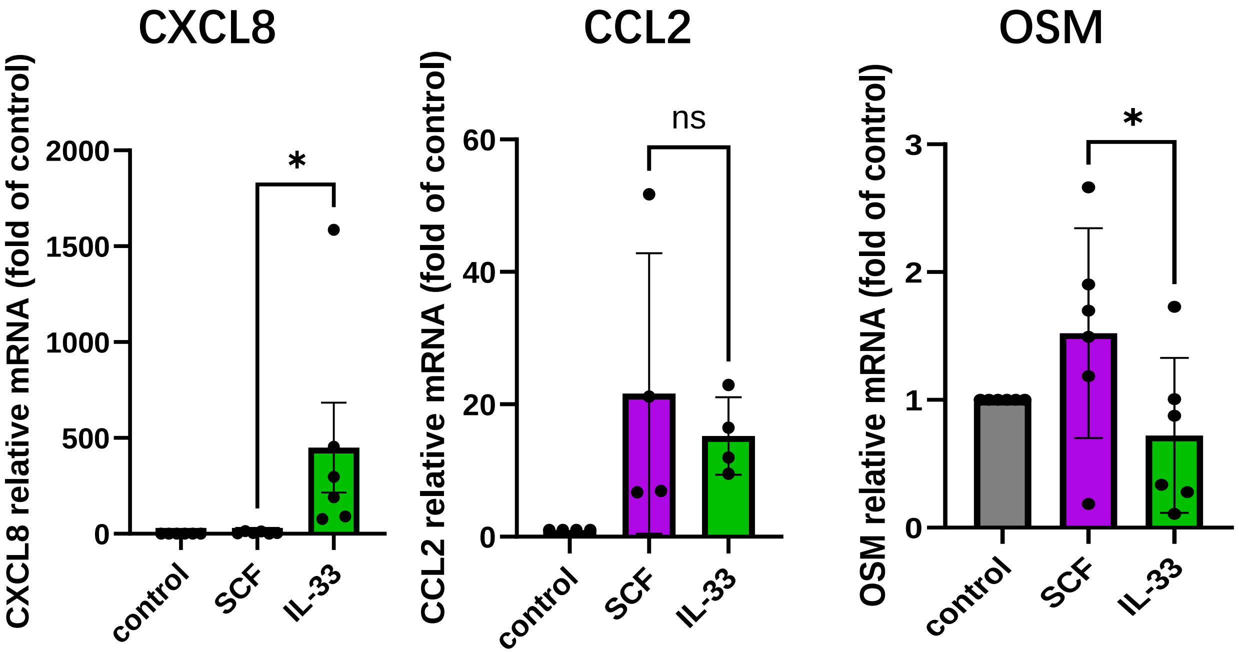

Figure S2: **IL-33 and SCF differentially regulate cytokine production in skin MCs.** mRNA levels of *CXCL8*, *CCL2* and *OSM* were assessed by RT-qPCR following stimulation with IL-33 (green) or SCF (purple) for 90 minutes. Expression was normalized to three housekeeping genes (*ACTB*, *GAPDH*, and *PPIB*) and is presented relative to the unstimulated control. The data are presented as individual dots with mean ± SEM. *$p < 0.05$ for SCF versus IL-33; ns: not significant.

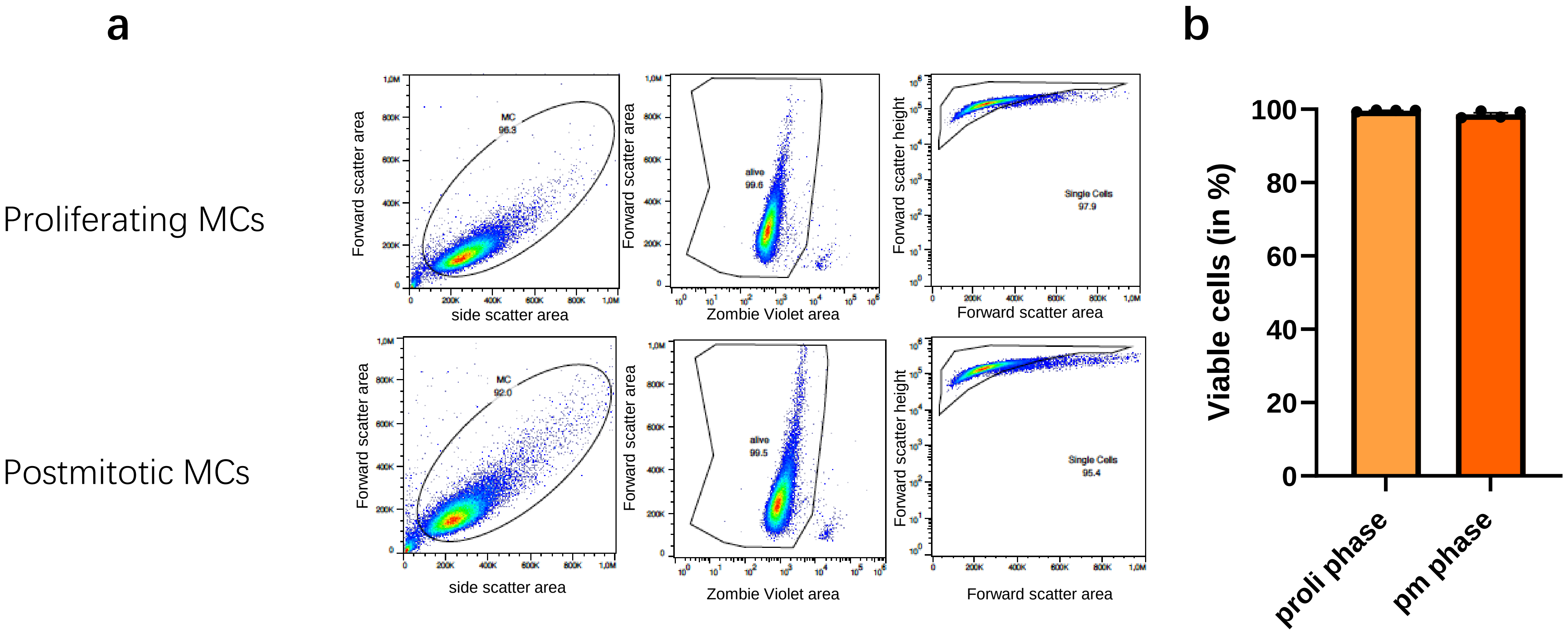

Figure S3: **Proliferating and postmitotic MCs are characterized by high viability**. (a) Representative flow cytometry dot plots showing the gating strategy and Zombie Violet live/dead staining. Cells negative for Zombie Violet were considered viable. Left: SSC/FSC staining, middle: Zombie Violet, right: single cells. (a) Cumulative data of n=4 experiments given as mean±SEM.
